# Sodium-Controlled Interfacial Resistive Switching in Thin Film Niobium Oxide for Neuromorphic Applications

**DOI:** 10.1021/acs.chemmater.4c00965

**Published:** 2024-05-31

**Authors:** Benedetta Gaggio, Atif Jan, Moritz Muller, Barbara Salonikidou, Babak Bakhit, Markus Hellenbrand, Giuliana Di Martino, Bilge Yildiz, Judith L. MacManus-Driscoll

**Affiliations:** † Department of Materials Science and Metallurgy, University of Cambridge, 27 Charles Babbage Road, Cambridge CB3 0FS, U.K.; ‡ Electrical Engineering Division, Department of Engineering, University of Cambridge, JJ Thomson Avenue, Cambridge CB3 0FA, U.K.; § Thin Film Physics Division, Department of Physics, Chemistry and Biology (IFM), Linköping University, Linköping SE-581 83, Sweden; ∥ Department of Nuclear Science and Engineering, Massachusetts Institute of Technology, 77 Massachusetts Avenue, Cambridge, Massachusetts 02139, United States; ⊥ Department of Materials Science and Engineering, Massachusetts Institute of Technology, 77 Massachusetts Avenue, Cambridge, Massachusetts 02139, United States

## Abstract

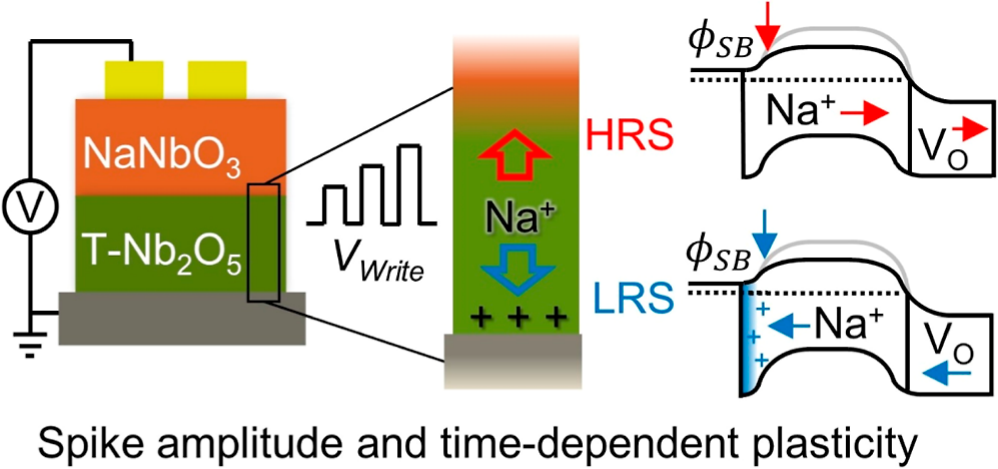

A double layer 2-terminal device is employed to show Na-controlled interfacial resistive switching and neuromorphic behavior. The bilayer is based on interfacing biocompatible NaNbO_3_ and Nb_2_O_5_, which allows the reversible uptake of Na^+^ in the Nb_2_O_5_ layer. We demonstrate voltage-controlled interfacial barrier tuning via Na^+^ transfer, enabling conductivity modulation and spike-amplitude- and spike-timing-dependent plasticity. The neuromorphic behavior controlled by Na^+^ ion dynamics in biocompatible materials shows potential for future low-power sensing electronics and smart wearables with local processing.

## Introduction

The rapid increase in demand for data-hungry technologies has led to a corresponding rise in the electricity usage of data centers.^1,2^ As we reach the limits of device scaling (i.e., Moore’s law), improvements in hardware efficiency beyond traditional von Neumann architecture become vital to enable data-hungry computation.^3^ Taking inspiration from the synaptic modulations in the brain, memristive devices have attracted interest for application towards high-density integration and analog processing, where nonvolatile resistive states are programmable via electrical bias.^4−8^ Hardware efficiencies are also needed for the Internet of Things (IoT) devices, which are currently limited by the power consumption from continuous data storage and communication.^9^

One type of emerging memristive technology is based on the resistive switching (RS) mechanism. Most RS technologies rely on redox changes, as the migration of ionic species controls the electronic conductivity of the metal oxide switching layer,^10,11^ with different functional materials having been explored for memristive applications.^12−14^ In filamentary devices, charged species migration can form/rupture filaments that determine the resistance state.^15,16^ However, when ionic migration occurs homogeneously across the device, it may result in gradual, controlled modulation of electronic barriers at thin film interfaces.^17^ Interfacial switching mechanisms offer the potential advantage of better controllability, low stochasticity, and high endurance^18−20^ compared to filamentary switching. Often, oxygen vacancies are the ionic defects that are involved in both filamentary and interfacial switching.^21,22^ Switching with small cations has also been studied, predominantly focusing on high-mobility species like protons,^23−25^ or Li^+^ ions.^26,27^

Moreover, there have been promising reports employing interfacial control of Na^+^ in TiO_2_ with neuromorphic applications.^28^ From a switching device perspective, Na^+^ offers advantages over Li^+^ and H^+^ for conductance modulation, such as lower flammability than Li^+^ and long-term stability than Li^+^ and H^+^ due to its larger ionic radius.^29^ In addition, from a biological perspective, Na^+^ is a common biomarker that plays a significant role in synapses, as neural networks operate by the motion of Na^+^, K^+^, and Ca^2+^ transport across membranes through interfacial processes.^30−32^

Beyond traditional computing and CMOS-applications, neuromorphic computing is a critical component to achieve edge computing and reduce power consumption in IoT or smart wearables.^19,33−36^ On top of reducing power consumption, edge computing has the potential to reduce device complexity, remove wireless range and time latency limitations, and data privacy issues related to wireless data transfer.^37^ The achievement of ionically controlled neuromorphic processing, where sensed ions are the active drivers of neuromorphic computation, enables response and interpretation of environmental stimuli in a single integrated device, desirable for interfacing with biological systems.^38,39^

T-Nb_2_O_5_ (“T” denotes the orthorhombic phase) is a widely studied anode material for energy storage applications due to its fast ion diffusion kinetics,^40^ as demonstrated in Li-^41−43^ and Na-ion batteries.^44,45^ Compared to other memristive materials, like TiO_2_ and WO_3_^23,29^ that have been used in ionically driven switching, T-Nb_2_O_5_ offers distinct ionic transport properties that have not been explored for this purpose. Niobium oxides are employed in commercialized Li-ion batteries, showcasing the versatility of this material in the orthorhombic phase for industrial and scalable use. Despite this, previous investigations of niobium oxides’ memristive properties have mainly focused on oxygen vacancy filamentary^21,46−51^ and threshold switching functionality^52−54^ without demonstrating evidence for synaptic plasticity. There have been reports of Li^+^ filamentary-based switching in LiNbO_3_,^55−57^ but the neuromorphic properties or electrical robustness of these processes remain uncharacterized, despite reported intercalation dynamics of Li-ions.^58,59^

We report neuromorphic performance based on interfacial switching modulated by the motion of Na^+^ ions, specifically, the modulation of the interfacial Schottky barrier in a NaNbO_3_/Nb_2_O_5_ solid-state 2-terminal device. Biological synaptic plasticity is shown for both spike-amplitude- and spike-time-dependent measurements controlled by Na^+^ dynamics. In this work, a NaNbO_3_ layer is grown atop epitaxial Nb_2_O_5_ to serve as a source for Na^+^. NaNbO_3_ is a stable perovskite phase that has variable Na stoichiometry on the A site, which has been shown to accommodate at least 2% A-site deficiency.^60^ Consequently, this is sufficient to dope the underlying Nb_2_O_5_ layer by Na^+^ reversibly as a function of bias. Both Na and O exchange is possible between the two layers, but we show via operando Raman methods that, thanks to the fast cation intercalation in T-Nb_2_O_5_, the Na^+^ exchange dominates the conductivity modulation in the switching and neuromorphic behavior reported here. We show that the bilayer structure is critical to achieving nonvolatile resistive states and synaptic plasticity functionality for neuromorphic applications. Furthermore, Nb_2_O_5_ thin films have been widely investigated in flexible electronics and biocompatible applications.^34,61−63^ Na^+^ switching enables synaptic learning ability in Nb_2_O_5_, offering new possibilities for smart sensing device applications.

## Materials and Methods

### Fabrication

Nb_2_O_5_ targets are prepared by ball milling amorphous Nb_2_O_5_ powder (99.9% trace, Sigma-Aldrich, UK) in ethanol, pelletizing, and sintering at 1073 K for 12 h. NaNbO_3_ targets are prepared by ball milling amorphous Nb_2_O_5_ powder and Na_2_CO_3_ (99% trace, Sigma-Aldrich, UK) in ethanol in a 1:1 Nb/Na molar ratio, pelletizing, and sintering at 1073 K for 12 h. Films were grown on conductive single-crystal Nb-doped strontium titanate (NbSTO) substrates in the (110) orientation by pulsed laser deposition (PLD) using a KrF excimer laser with a 248 nm wavelength. The deposition chamber was evacuated to sub 1 × 10^–5^ mbar, and the oxygen partial pressure during growth was kept constant at 1.3 × 10^–2^ mbar. The bilayer devices are grown without breaking a vacuum. Both bilayer and single-layer devices are grown at 620 °C, with 0.9 mJ/cm^2^ laser fluence, and a shot frequency of 4 Hz. Single-layer devices are 50 nm in thickness; the total thickness of the bilayer device is also 50 nm, with 25 nm thick Nb_2_O_5_ and 25 nm thick NaNbO3. For electrical characterization, the films are patterned by UV lithography to obtain circular electrode pads of diameter 100, 50, 25, and 20 μm. Cr/Au (6/60 nm in thickness) top electrodes (TEs) are deposited by electron-beam evaporation using a PVD 200 Pro (Kurt J. Lesker) instrument.

### Materials Characterization

Cross-sectional transmission electron microscopy (TEM) analysis was performed on an FEI Tecnai Osiris microscope operated at 200 keV. Z-contrast images were acquired in scanning TEM high-angle annular dark-field (STEM-HAADF) mode. Energy-dispersive X-ray (EDX) elemental line scans were also obtained by employing a FEI Super-X spectrometer embedded in the FEI instrument. The TEM specimen was prepared by the focused ion beam (FIB) technique using a FEI Helios NanoLab microscope. The elemental compositions and depth profiles of the oxide thin films were obtained using time-of-flight elastic recoil detection analysis (ToF-ERDA), which is a powerful standard-free tool for quantitative analysis and depth-profiling with high accuracy, in particular, for light elements including Na and O. ToF-ERDA was carried out in a 5-MV 15SDH-2 tandem accelerator where recoils were detected at an angle of 45° with respect to the primary beam in a telescope measuring time-of-flight (ToF) using a foil-detector and energy in a gas ionization chamber in coincidence. This approach provides mass-resolved data in ToF-vs-energy plots. Recoils were created by employing a 36-MeV ^127^I^8+^ beam incident at 67.5° with respect to the surface normal of the samples. The depth profiles and average elemental compositions were determined from ToF-ERDA time-energy coincidence spectra using Contes and Potku software packages. Raman spectroscopy was acquired through a thin TE Cr + Au (3 + 6 nm) layer that was deposited by thermal evaporation^64^ using a 0.8-NA 100× objective from Olympus, with integration times of 10 s. The Raman signals were excited using a 633 nm C.W. single-longitudinal-mode laser manufactured by Integrated Optics and detected using a Kymera spectrometer that is connected to an Oxford Instruments Newton EMCCD camera. These measurements were collected on dedicated samples deposited on a lanthanum aluminate (LAO) (110)/lanthanum strontium manganate (LSMO) (10 nm) substrate of double thickness (50 nm Nb_2_O_5_ + 50 nm NaNbO_3_), chosen to the strong Raman activity of NbSTO and improve film intensity.

### Electrical Characterization

Current–voltage characteristics and spike measurements were investigated by using a Keysight B2912A source measure unit and a probe station. Electrochemical impedance spectroscopy (EIS) is measured employing a Solartron impedance analyzer, with frequencies ranging from 1 MHz to 1 Hz and an ac-voltage amplitude of 100 mV. In all electrical measurements, the bottom electrode (BE) is grounded, and the amplitude of read voltages is maintained at 0.1 V, including for ac-voltage EIS measurements.

## Results and Discussion

We begin by presenting the structural and compositional information on NaNbO_3_/Nb_2_O_5_ bilayer devices. We then show their electrical performance and compare these with devices from the individual layers. Next, we explore the bilayer neuromorphic performance by studying spike-amplitude- and spike-timing-dependent plasticity. In situ Raman spectroscopy was employed to probe the critical role of Na^+^ in the RS.

**Figure 1:**
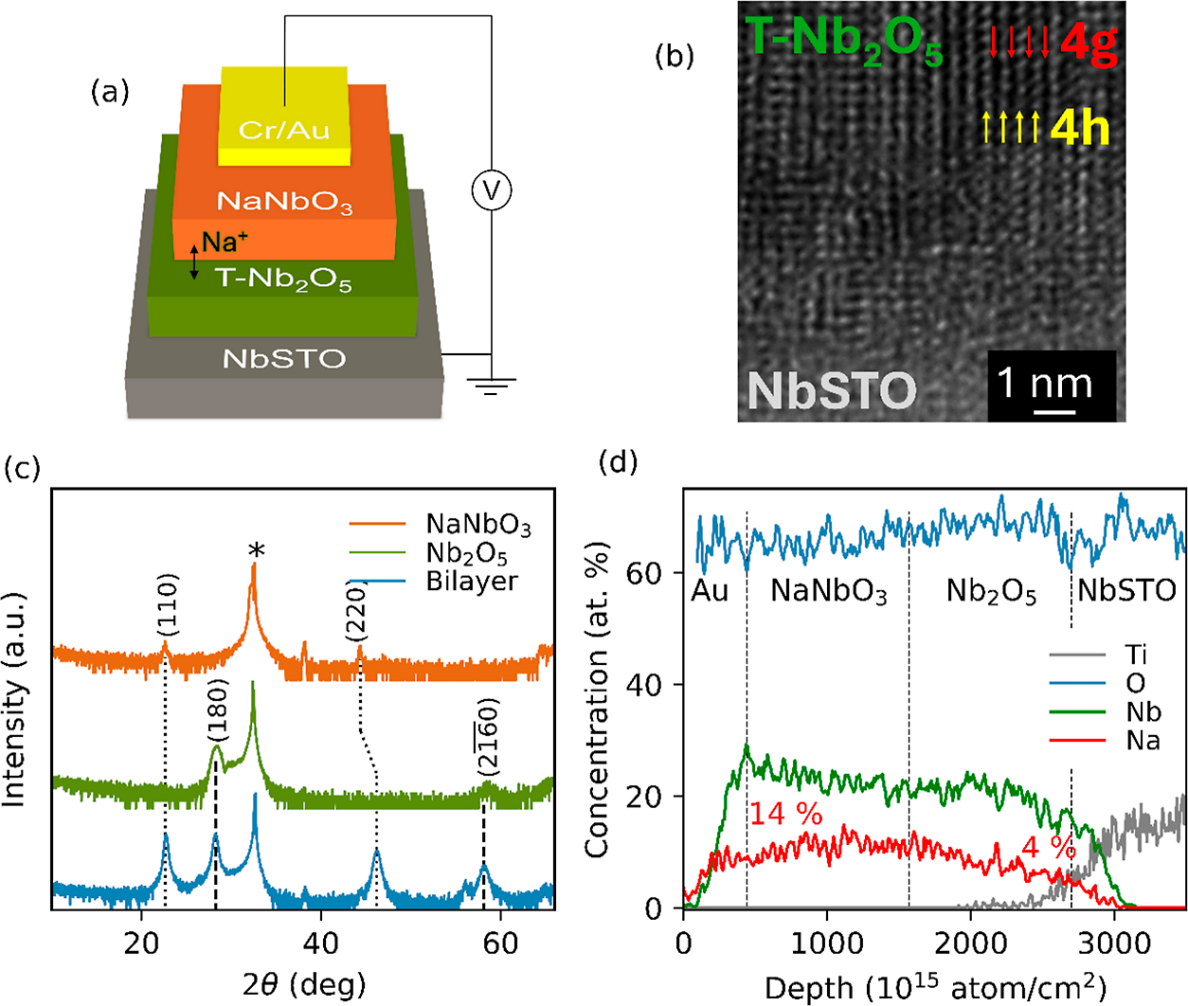
“(a) Schematic representation of bilayer structure used in this work: (110) NbSTO substrate/T-Nb_2_O_5_/NaNbO_3_/Cr/Au. (b) Cross-sectional HRTEM of device in (a), highlighting the alternating 4h (yellow) and 4g (red) layers in T-Nb_2_O_5_ that create atomic column-like structures. (c) XRD spectra of NbSTO/NaNbO_3_ thin film (orange), NbSTO/T-Nb_2_O_5_ thin film (green), NbSTO/NaNbO_3_/Nb_2_O_5_ bilayer (blue), dotted-lines highlight overlap of identified phase peaks for each individual layer, substrate peak signaled by asterisk. (d) ToF-ERDA depth profiling of bilayer device, with elemental quantification and analysis, showing Na-gradient forming across bilayer device.”

Figure 1a schematically illustrates the bilayer structure of the NbSTO/Nb_2_O_5_/NaNbO_3_/Cr/Au stack. Figure 1b shows the cross-sectional HRTEM image of the T-Nb_2_O_5_ film with a dense nanostructure epitaxially grown on 110-oriented NbSTO. This nanostructure consists of alternating densely and loosely packed Nb-oxide polyhedral sheets (4h and 4g, respectively). Griffith et al. showed that the layered nanostructure in epitaxial T-Nb_2_O_5_ enables fast cation migration through the atomic column-like structure of low steric hindrance (low Nb-density).^41^ It was already reported that a (110) perovskite substrate is critical to achieve the perpendicular orientation of the 4h/4g columns with respect to the T-Nb_2_O_5_ plane.^58^

Figure 1c compares the XRD spectra of the bilayer (blue) to those of its constituent single layers, Nb_2_O_5_ (green) and NaNbO_3_ (orange). The diffraction peaks appeared at 2θ = 28.40° and 58.78° are attributed to the (180) and (2160) orientations of the large orthorhombic cell of Nb_2_O_5_ (T-phase, *a* = 6.175 A, *b* = 29.175 A, *c* = 3.920 A; α = β = γ = 90°), as previously reported.^65^ NaNbO_3_, deposited on top of T-Nb_2_O_5_, is in its orthorhombic phase (*Pmc*2, *a* = 5.53, *b* = 5.61, *c* = 7.79 A; α = β = γ = 90°), as determined by the XRD peaks at 2θ = 22.42° and 44.12°, attributed to (110) and (220) reflections in both the single layer (orange) and bilayer film (blue). The growth temperature is optimized to 620 °C to achieve the columnar structure of T-Nb_2_O_5_. This is below the ideal growth temperature for epitaxial NaNbO_3_ and, therefore, results in a polycrystalline layer, as evidenced from XRD.

A ToF-ERDA elemental depth profile of the pristine NbSTO/Nb_2_O_5_/NaNbO_3_/Cr/Au stack is given in Figure 1d. Both Nb and O have an almost uniform distribution along the Nb_2_O_5_/NaNbO_3_ bilayer. While Na is uniformly distributed along the thickness of the NaNbO_3_ top layer (with an average concentration of 14 at. %), it has a gradient in the Nb_2_O_5_ bottom layer, significantly decreasing from 14 at. % (the Nb_2_O_5_/NaNbO_3_ interface) to 4 at. % (the NbSTO/Nb_2_O_5_ interface). This indicates the high diffusivity of Na in the Nb-based oxides that can provide versatile cation mobility upon applying bias, which is essential for low-power RS. To show the challenge of Na-doping the Nb_2_O_5_ layer in PLD, 5 films deposited from targets of varying Na/Nb ratios (1, 0.8, 0.5, and 0.2) are compared (XRD, Supporting Information Figure S2). It was found that only the targets closest to the precise NaNbO_3_ stoichiometric ratio led to the observation of NaNbO_3_-related peaks. To confirm the greater stability of stoichiometric NaNbO_3_ versus Na-deficient NaNbO_3_, ToF-ERDA analysis was further performed on these samples (Supporting Information Figure S2). The presence of Na is observed only for the stoichiometric case. As already mentioned, the stability of the top cation source is critical to its choice as a controlled Na source for the doping of the Nb_2_O_5_ host.

**Figure 2:**
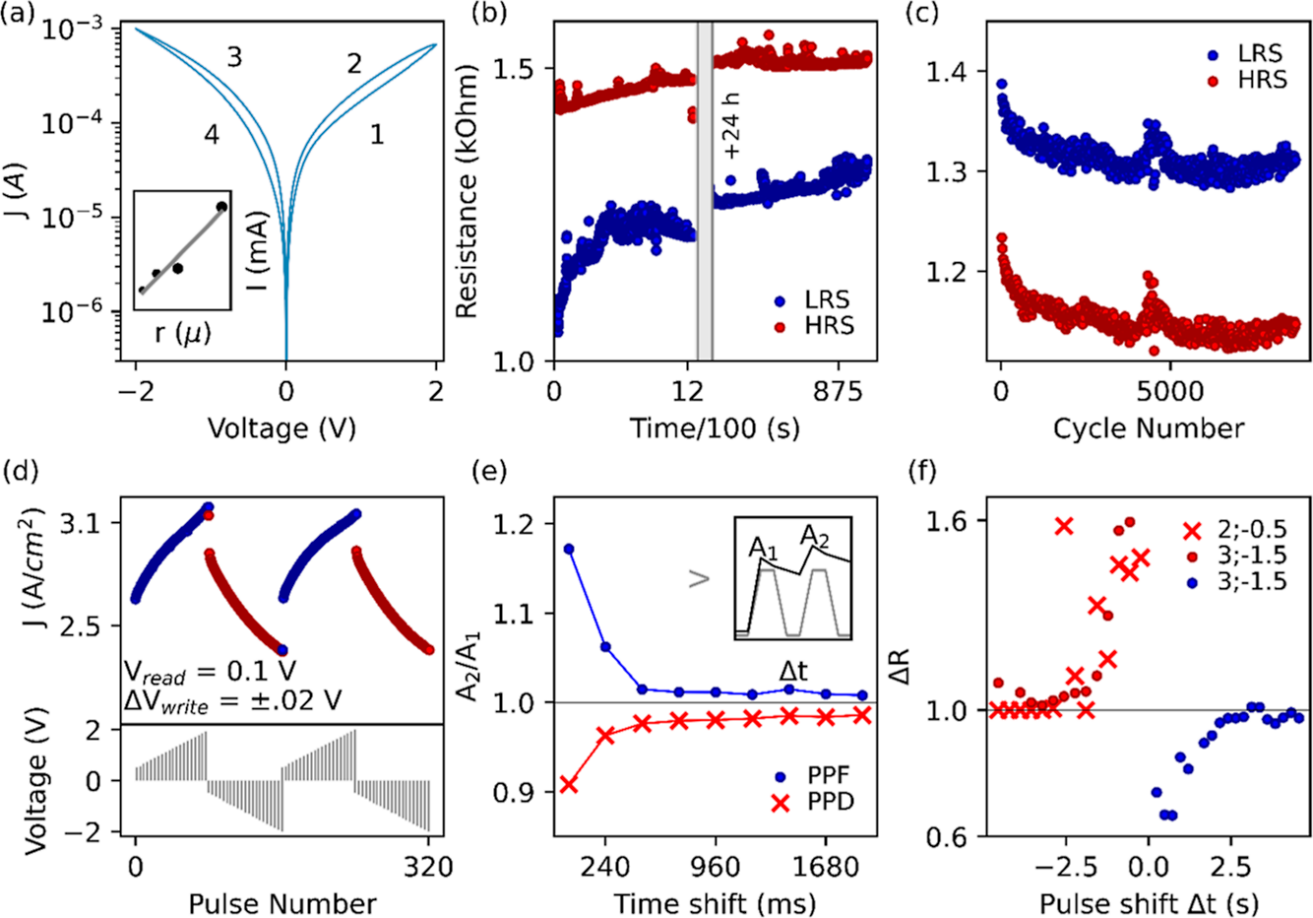
“(a) *I*–*V* characteristics of bilayer device with CCW direction of RS; inset presents area-dependent current. (b) Retention plots for bilayer device showing nonvolatile HRS/LRS after ±2 V set/reset pulses (red and blue, respectively) read at 0.1 V per second for 1000 s; the measurement is then repeated for LRS and HRS on the same respective devices after 24 h. (c) Endurance plots for 8000 RS cycles on bilayer device after ±2 V set/rest pulses, read at 0.1 V. (d) Potentiation and depression of device synaptic weight via increasing train pulses from 0.5 to 2, in Δ*V* = ± 0.1 V with 0.1 V pulse read, inset showing a schematic of voltage profile. (e) (PPF, red) and depression (blue) using two consecutives +2 and −2 V pulses, respectively, showing the A2/A1 index ratio as a function of pulse interval time. (f) Asymmetric STDP demonstrating learning ability in device resistance potentiation and depression as a function of pre- and postsynaptic voltage pulse interval time.”

The device was contacted via a Cr/Au TE, achieving a 2-terminal configuration, ideal for its simplicity.^66^ Since the electrical bias is applied parallel to the T-Nb_2_O_5_ channels, this creates a source/host structure for the charge carrier species to exchange under the bias application at the TE and grounding at the BE, NbSTO. Figure 2a shows the characteristic RS counterclockwise pinched loop, showing set/reset transitions at ±2 V, which switch the device between the low resistive state (LRS) and high resistive state (HRS) in a bipolar fashion. The switching process is forming-free and gradual, ideal features for analog artificial synapses candidates.^67^ The current is found to be linearly proportional to electrode pad size (10, 12.5, 25, and 50 μm radii, Figure 2a inset), which is evidence for interfacial switching and not a filamentary-dominated mechanism, and this is in agreement with previous results reported for Schottky barrier devices.^18^ On the contrary, filamentary Nb_2_O_5_ switching devices show abrupt jumps in current and filament formation/rupture events,^51^ which limit the uniformity from cycle to cycle and across devices. Uniformity is a crucial requirement for RRAM-based synaptic devices, affecting training accuracy, power consumption, and overall performance efficiency of the device at a large-scale. Here, we focus on cycle-to-cycle uniformity on a single device batch (Supporting Information Figure S3); however, to bring this material and device structure closer to application, future work should investigate uniformity across batch depositions, employing practical fabrication methods that enable large-area deposition (see conclusion for further discussion on PLD and other fabrication methods for niobium oxides).

To assess the retention performance, the device is set with a pulse of 2 V (and reset with −2 V) for 1 s, and the resulting resistance states are read with a pulse of 0.1 V (Figure 1b) for 1000 pulses at 1 s intervals. The positive pulse sets the device into the LRS, which increases by 12% of its initial resistance in the first 1000 s, followed by a plateau. The LRS is conserved after 24 h with a further 4% increase. The negative pulse resets the device to the HRS, stable within 6% of its initial state for the first 1000 s and stable after 24 h. In Supporting Information Figure S4, we compare the retention for bilayer and single layer devices: notably, nonvolatile behavior is observed only for the bilayer, while the single layers decay back to their initial state. Importantly, using the NaNbO_3_ as a Na-doping source in combination with the ion conducing Nb_2_O_5_ is critical for achieving the nonvolatile RS performance studied herein. Hence, the interfacing of a stable Na-source with Nb_2_O_5_ enables conductance tuning as a response to an applied field, showing the sensing behavior of the device stack. The integrated ionic monitoring and memristive functionality can have interesting applications toward sensing edge computing usage cases, such as biocompatible monitoring. It is noteworthy that the ionic exchange between Nb_2_O_5_ and NaNbO_3_ could be expected to involve both Na^+^ and oxygen species under the same bias polarity; however, we suggest that, thanks to the faster mobility of Na-cations in T-Nb_2_O_5_, the Na^+^ exchange dominates the conductivity modulation. We further employ operando Raman and temperature-dependent measurements to show Na-specific chemical composition modulations.

The device endurance is demonstrated in Figure 2c by applying ±2 V write pulses of 25 ms duration and 0.1 V reading pulses. The device shows uniform and reliable cyclability across >8000 cycles, maintaining a stable memory window and IV sweep response (Supporting Information Figure S3c). The retention and endurance performances are fit for both short- and long-term learning operations.^68^ The memory window is tunable depending on the amplitude and time of the pulse train, as demonstrated further in the neuromorphic characterization section. This functionality can be applied toward translating stimuli sensing to a resistance state. The conductance modulation shows promise toward multilevel-state performance, which is out of the scope of this investigation but is promising behavior for ultralow power neuromorphic and biosensing applications. The on/off ratio maximum is found to be 3.6 (Supporting Information Figure S4), lower than generally reported values^33^ but compliant with neuromorphic operation requirements.^69,70^ It is noteworthy to mention that Nb metal oxide systems with Li^+^ ion intercalation have been reported with large conductance changes based on a different mechanism: the oxide undergoes an insulator-to-metal transition (IMT) due to the formation of the LiNbO_3_ phase and population of the Nb-center conduction band.^58^ Also, previous reports have shown analogue behavior in Nb_2_O_5_ controlled by an electronic charge trapping mechanism; however, the behavior is limited by poor retention.^71^ On the contrary, the nonvolatile analogue and interfacial switching nature of the bilayer device reported here enables neuromorphic and sensing applications, as we further explore in the next section.

Neuromorphic devices with sensory functions have the capacity to process information from the perceived surroundings at ultralow power by emulating neural learning functionality.^33,72^ On top of ionic sensitivity, materials for neuromorphic applications must, therefore, be able to satisfy some key functionalities. Synaptic plasticity is the ability of a synapse to modulate its weight, which in turn determines the efficiency with which adjacent neurons are able to propagate information among each other, represented here as the device resistance.^73^ Plasticity can be differentiated into long-term and short-term forms (LTP and STP, respectively). LTP denotes a nonvolatile state equivalent and in other words, underpins the formation of long-term memory in learning functions–hours to years. STP refers to temporary functions in shorter time windows—milliseconds to minutes—to enable tasks such as various stimuli recognition, filtering, and perception.^74^ In the peripheral nervous system, for example, STP functionality enables stimuli detection and information processing on different sites without relying on relaying information to the central nervous system. In analog sensor devices, STP can be employed to perform brain-inspired algorithms where recent neural activity has to be tracked.^37^ In the brain, the action potential has been found to have five orders of complexity, i.e., state variable dependencies that determine the electrical spike profile taking place across neurons and synapses.^75,76^

Figure 2d shows spike amplitude-dependent plasticity (SADP), investigated by application of a train of pulses of increasing voltage, from 0.5 to 2 V (and −0.5 to –2 V), with Δ*V* = ± 0.1 V, and each pulse followed by a 0.1 V read pulse (Figure 2d, bottom inset). The device resistance is tuned by the amplitude of the write pulse, showing alternating potentiation and depression of the resistance state, where each data point represents the average readout after each pulse has been applied. This effect is analogous to synaptic weight plasticity and is found to be reversible, resilient to cyclability, and uniform across different devices tested (Supporting Information Figure S5). We demonstrated 80 states each per potentiation and depression, with an average percentage change in resistance of 0.20 and −0.27%, respectively. These values are in line with similar ionic systems, such as protonic conductance changes in WO_3_ systems.^23^

Next, we investigate the role of spike-timing in the modulation of synaptic weights (Figure 2e,f). First, STP learning behavior is investigated by performing paired-pulse facilitation (PPF): two consecutive pulses are applied to the bilayer, alternating potentiation (+1 V) and depression (−1 V) at increasing time shifts (from 240 ms to 2 s) (Figure 2e). The ratio between the amplitudes of the two current peaks, defined as postsynaptic spikes A1 and A2, is then plotted as a function of pulse interval.^74^ In agreement with the STP behavior observed in short-term memory and neural network mechanics, the postsynaptic ratio is dependent on the pulse time interval, showing a greater change for closely timed pulses. The frequency dependence observed in neuromorphic devices has been previously related to intrinsic switching mechanism time constants: when the pulse pair interval is shorter than the mechanism relaxation dynamics, the nonequilibrium states are stimulated by the second pulse, and the change in conductance effect is maximized.

Second, we investigated the modulation of synaptic weight as well as direction (potentiation or depression) by the temporal order, or time shift, between pre- and postsynaptic spikes. Pre- and postsynaptic pulses are simulated at time intervals (from 240 ms to 5 s) and summed so that the overall voltage is applied at the TE; the pulses are asymmetric (Supporting Information Figure S6). By negative time interval, we denote the pulse in the order pre → postsynaptic and positive as pulses being post → presynaptic. Each response is measured after applying a set (or reset) pulse of constant amplitude to bring the device back into its LRS (HRS). In Figure 2f, the bilayer changes in resistance, |Δ*R*|, as a function of time intervals, indicating an inverse proportionality to time shift. This behavior has been previously associated with asymmetric Hebbian learning and has potential for applications in spiking neural network systems, where the frequency of a train of pulses is converted into a response of proportional intensity.^77^ In both time-dependent measurements, PPF and STDP, the time intervals investigated for synaptic weight modulation are approximately 1 order of magnitude slower than biological time scales.^78,79^ However, this limitation is introduced by the experimental setup rather than the actual devices, thus still showing promising use of Na^+^ ion dynamics in biocompatible electronics. Switching speed is a meaningful parameter when evaluating the performance of devices for artificial neural network (ANNs) applications, as it enhances fast training and operational efficiency. The scope of this work is to report on the successful control of synaptic plasticity in Na-doped systems. However, future work should further explore how to translate the fast-charging performance of the columnar structure of T-Nb_2_O_5_ studied in energy storage applications towards the RS speed performance for neuromorphic devices.

**Figure 3:**
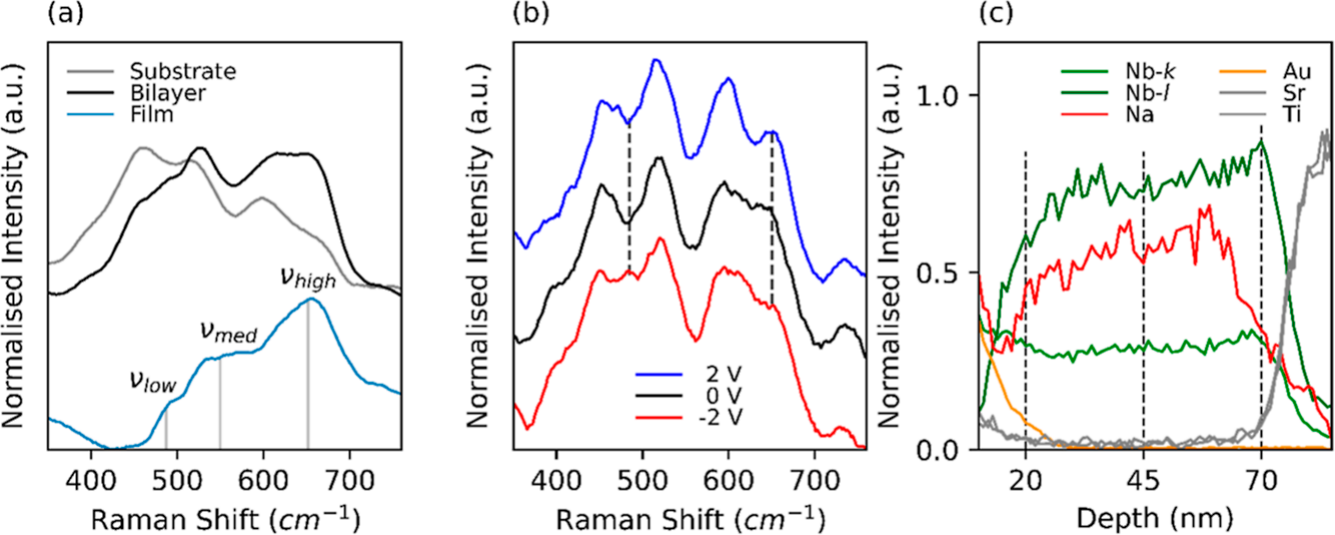
“(a) Raman spectroscopy measurement for LAO/LSMO substrate (gray) and LAO/LSMO/Nb_2_O_5_/NaNbO_3_ bilayer device (black), the active film Nb_2_O_5_/NaNbO_3_ spectra (blue) is obtained by subtraction of the substrate spectra. (b) In-operando Raman measurements of bilayer device, showing changes between pristine bilayer spectra (black) and after bias application: LRS after +2 V pulse (blue) and HRS after −2 V pulse (red). (c) EDX measurements on a cross-sectional sample measured by STEM (HAADF) in its LRS, showing Na-signal accumulation toward the Nb_2_O_5_/NbSTO interface.”

The modulation of interfacial energy barriers in the bilayer device was further probed by operando Raman spectroscopy to show the voltage-dependent migration of Na^+^ cations in the device. A bilayer film was grown on an LAO substrate with an 8 nm LSMO layer as conductive back contact, as the NbSTO substrate (employed in the default bilayer devices described in the rest of the paper) is highly Raman active and obscures the film signal. The Raman plot of the full bilayer stack is shown in Figure 3a; the strong peak around 600 cm^–1^ is attributed to the LAO/LSMO signal.^80^ To further isolate the Nb_2_O_5_/NaNbO_3_ active layer contribution, the Raman signal of the bare substrate LAO/LSMO (Figure 3a, gray) is subtracted from the full bilayer stack (LAO/LSMO/Nb_2_O_5_/NaNbO_3_) signal (Figure 3a, black. The Nb_2_O_5_/NaNbO_3_ Raman spectra (Figure 3a, blue) show a prominent peak ν_high_ ≈ 655 cm^–1^, accompanied by a shoulder ν_med_ ≈ 540–560 cm^–1^, and a smaller shoulder ν_low_ ≈ 460–490 cm^–1^. The results closely correspond to the NaNbO_3_ literature, attributing the high shift ν_high_ and medium shift ν_med_ regions to the NbO_6_ octahedra symmetric and asymmetric stretches in ANbO_3_ (A = Li, Na, and K) compounds, while low shift region ν_low_ is associated with O–Nb–O bending modes in ANbO_3_ compounds.^81,82^

Figure 3b shows the in-operando characterization of the LAO/LSMO/NaNbO_3_/Nb_2_O_5_ device, wherein Raman spectra were acquired concurrently under bias switching between ±2 *V*. The signal from LAO/LSMO is not subtracted here as it is expected to have bias-dependencies. The in-operando measurement enabled the study of the immediate effect of the applied bias on Na-intercalation, and changed in the device chemical. Figure 3b reveals the changes in Raman spectrum shape and intensity between the pristine state and HRS (red) and LRS (blue). At + 2 V (Na^+^ ions move away from TE), a rise in peak intensity is observed in the high wavenumber region at 655 cm^–1^, compared to the pristine state (at 0 V), while at −2 V (Na^+^ ions move toward TE), the intensity of this peak is reduced. The low wavenumber region, 450–490 cm^–1^, is also voltage-dependent, showing increased signal intensity upon −2 V pulse application. Chen et al. previously investigated the correlation between T-Nb_2_O_5_ Raman spectra and cation intercalation. The study showed that, upon deintercalation, the high wavenumber peaks increase due to reduced coordination between oxygen and cation species and enhanced stretching modes observed.^40^ Moreover, the low wavenumber peaks representing the bending vibration are expected to decrease due to the compression of NbO_*x*_ polyhedral from A-site vacancies in ANbO_3_.^60^ Therefore, operando Raman results observed in the bilayer device closely match the Na-intercalation effects previously reported in the literature, confirming that the applied bias drives the cation (de)intercalation in the device, across the Nb_2_O_5_ and NaNbO_3_ layers.

To investigate this phenomenon further, the operando measurements were repeated on the undoped Nb_2_O_5_ single layer device, where oxygen vacancies could be expected to accumulate at interfaces in response to the applied bias. Supporting Information Figure S7 shows that no change is observed in the high shift region at 655 cm^–1^, which allows us to attribute the changes observed in Figure 3b to voltage-controlled Na^+^ migration. In fact, it is well-known that small cations benefit from low steric hindrance in the T-Nb_2_O_5_ channeled layer and so fast ionic migration, which we further address in the next section via temperature-dependent measurements. Moreover, the polarity of potential applied was reversed (Supporting Information Figure S7), and the opposite trends compared to Figure 3b were observed, verifying the reliability of the measurement.

Cross-sectional scanning transmission electron microscopy (STEM) bright-field images of the bilayer device are presented in Figure 3c. The chemical composition of the cross-section was investigated by energy dispersive X-ray (EDX) spectroscopy. More precisely, a device previously set to its LRS was investigated ex situ to study the nonvolatile distribution of the Na^+^ ions. We observe a Na-species gradient that increases by 25% from NaNbO_3_ to Nb_2_O_5_, with a higher concentration found at the Nb_2_O_5_/NbSTO interface. Thus, the measurement confirms that setting the device via a positive pulse leads to the nonvolatile accumulation of the Na-species away from the TE. In comparison, the ToF-ERDA depth profile presented in Figure 1d shows that, in a pristine HRS device, the Na-species gradient *decreases* from NaNbO_3_ to Nb_2_O_5_.

**Figure 4:**
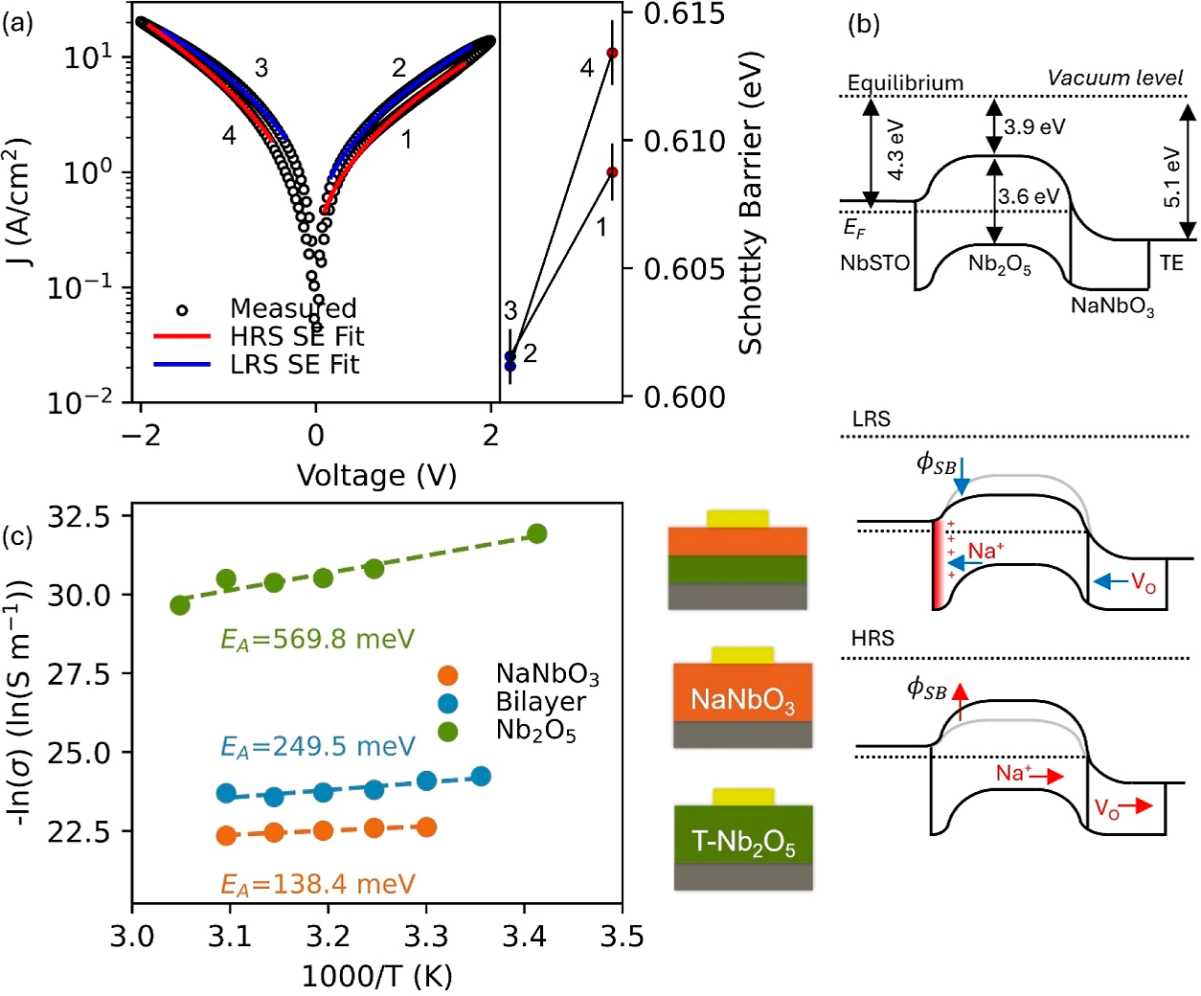
“(a) Left: Schottky emission fit over measured IV sweep data; right: Schottky barrier modulation upon set and reset voltage pulse operations. (b) From top to bottom: band diagram model for equilibrium, LRS, and HRS. (c). Left: Arrhenius derived conductivities versus  showing activation energies for bilayer device (blue), Nb2O5 (green), and NaNbO3 (orange) individual layer devices; right schematic cross-sectional models for systems studied in temperature-dependent derivation.”

Figure 4a shows a representative IV curve fitted with a Schottky emission mechanism that provides a consistently low error fit across the midvoltage range of the IV scan (i.e., 0.2–2.0 V), suggesting that it represents the dominating mechanism at play. The Schottky barrier (SB) is extracted according to (eq 1)
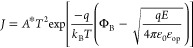
1where *J* is current density (A/cm^2^), A* = λ*A*_0_ is the effective Richardson constant, taken to be *A*_0_ = 1.2 × 10^6^ (A/m^2^ T^2^), λ = 0.5, *T* = 291 is the temperature (Kelvin), ε_0_ is the permittivity of vacuum, Φ_B_ is Schottky barrier (eV), and ε_op_ is the optical dielectric constant, both to be calculated. Figure 4b compares the SB at various points of the IV curve to show the quantitative change, ΔSB, found to be ±0.015 across set/reset. We note that similar changes in SB were reported for an interfacial Na-based system in 2-terminal Na-doped TiO_2_ devices, where Na-ions are the main switching charge carriers.^28^ We suggest that because of the large doping present in the Nb_2_O_5_ layer, the Na-cation gradient formed at the interfaces after electrical pulsing is limited, leading to smaller SB changes and overall smaller on/off ratios observed compared to the aforementioned Na-doped titania devices. However, neuromorphic functionality is still shown, and in small amplitude ranges, compatible with sensing applications.^68^

A temperature-dependent fit of thermionic emission^83^ was then performed to sustain the voltage-dependent derivation presented above of the Schottky mechanism (Supporting Information note 8). The Richardson plot derivation of the SB shows good agreement with the voltage-dependent method described above, consolidating the suitability of Schottky emission as the dominating conduction mechanism to describe the behavior observed.

We can understand the resistive switching and neuromorphic behavior shown in Figure 2a–f based on the homogeneous infiltration of sodium in the Nb_2_O_5_ thin film (Figure 1b,c) and its voltage control (Figure 3a–c). In the two-terminal device configuration, the column-like structure of the T-Nb_2_O_5_ lies perpendicular to the plane and parallel to the applied bias, enabling voltage-controlled migration of Na-species. IV (Supporting Information Figure S4) and EIS measurements (Supporting Information Figure S9) show that the as-grown NaNbO_3_ layer (*R*: 200 Ω) is more conductive than the Nb_2_O_5_ single layer (*R*_HRS_: 1.5 × 10^3^ Ω) and the Nb_2_O_5_/NaNbO_3_ bilayer device (*R*: 8.2 × 10^7^ Ω). Thus, Nb_2_O_5_ is the active switching layer, whereas NaNbO_3_ is passive to the switching but is critical as a stable Na-source. For consistency, we therefore use only the Nb_2_O_5_ thickness in the calculations of the electric field presented here and refer to the NaNbO_3_/TE interface as Ohmic.

In the positive voltage sweep applied at the TE, the Na^+^ species accumulate at the NbSTO/Nb_2_O_5_ interface, which is the dominating conduction barrier in the case of a positive voltage at the TE. As the Na dopants act as electron donors due to the low ionization energy, the increase in Na-concentration at the interface reduces the Schottky barrier height, which sets the device to the LRS. With the application of the negative sweep at the TE, the Na^+^ ions move away from the Nb_2_O_5_/NbSTO interface, resetting the device to an HRS for the following positive voltage sweep. As mentioned earlier, it is possible that oxygen species also exchange across the Nb_2_O_5_/NaNbO_3_ interface: in a negative sweep, oxygen vacancies accumulating in the NaNbO_3_ would reduce the Schottky barrier at the Nb_2_O_5_/NaNbO_3_, which is the dominating conduction barrier for a negative voltage at the TE. Importantly, different from the IV sweeps, where the two Schottky barriers limit the current for positive and negative voltages at the TE, in the endurance/retention and neuromorphic measurements of Figure 2a–f, the read pulses are positive so that the Schottky barrier at the Nb_2_O_5_/NbSTO interface is the limiting barrier for both the HRS and LRS. Oxygen-vacancies have also been shown to enhance the Li-ion intercalation process^84^ in T-Nb_2_O_5_ systems.

Finally, the temperature-dependent ionic diffusion activation energy of the bilayer is compared to those of its constituent layers, more precisely, the Nb_2_O_5_ and NaNbO_3_ single layers. For each device, the EIS spectra are measured over a temperature range and fitted by an equivalent circuit (Supporting Information Note 10). The activation energy of diffusion (Figure 4c) is then derived from (eq 2)
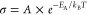
2where σ is conductivity (S/cm), *A* is the pre-exponential factor, *k*_B_ is the Boltzmann constant, *T* is the temperature (Kelvin), and *E*_A_ is the activation energy to conduction (eV). First, for the bilayer, we report an activation energy of 249.5 meV, in excellent agreement with literature Na-cation diffusion values in metal oxides and small cation diffusion reported for T-Nb_2_O_5_ systems studied previously.^85−88^ In comparison, the largest activation energy is obtained for the pristine Nb_2_O_5_ device, 569.8 meV. This value is in excellent agreement with previously reported oxygen vacancy activation energy in Nb_2_O_5_ thin films,^89−91^ which do not travel via the columnar-structure and are hence not kinetically facilitated by the orthorhombic phase. The values obtained indicate that the Na^+^ ion is the dominating charge carrier responsible for the interfacial switching in the bilayer device, as presented by our mechanism model.

## Conclusions

In conclusion, a double layer NaNbO_3_/Nb_2_O_5_ is studied to show the Na^+^ control of interfacial RS. The interfacing of these two layers enables uniform Na-doping, leading to voltage-controlled migration of Na^+^ species in the Nb_2_O_5_ layer, facilitated by perpendicularly grown columnar structures. Electrical characterization highlights formation-free and gradual RS, indicative of interfacial mechanisms, with robust retention. The device shows synaptic weight modulation by both spike-time and amplitude dependencies, characterized by SADP and STDP studies. In operando Raman spectroscopy elucidates the voltage-dependent migration of Na^+^ within the device, supporting a Na^+^-modulated conductivity. Additionally, EDX spectroscopy confirms the nonvolatile distribution of Na^+^ species in the device LRS configuration. We propose a Schottky emission mechanism controlled by Na^+^ dynamics as the primary charge carrier at the conduction barrier interfaces, driving the device between LRS and HRS under positive and negative voltage sweeps, respectively.

While PLD enables epitaxial thin film growth with high stoichiometric control, future work will explore the translation of this system to more practical fabrication to enable a larger area deposition methods, necessary for high-density crossbar array application. Nb_2_O_5_ thin film growth by sputtering or atomic layer deposition (ALD) on silicon is well-established for electronics applications, and the orthorhombic phase, critical for ionic intercalation purposes, has also been scaled up for the commercialization of Nb-based Li-ion batteries. However, the translation of the vertically oriented nanocolumns in T-Nb_2_O_5_ from single crystal substrates (i.e., NbSTO, LAO) to more practical substrates has not yet been yet explored.

## Supporting Information Available

Details of thin film surface morphology and composition studies; characterization of device uniformity in electrical and neuromorphic measurements; comparison of Na-doped and undoped sample performance; further electrical, in-operando material, and impedance characterization detail; and derivation of Schottky barrier (PDF)
