# Supplementary material for: Sodium-Controlled Interfacial Resistive Switching in Thin Film Niobium Oxide for Neuromorphic Applications
Source: Chem Mater. 2024 May 31;36(11):5764–74. doi: 10.1021/acs.chemmater.4c00965 (PMC11170940; doi:10.1021/acs.chemmater.4c00965)
Supplement: Supplementary file 1 — cm4c00965_si_001.pdf [file cm4c00965_si_001.pdf]

Supplementary Information

Sodium-controlled interfacial resistive switching in thin film niobium oxide for neuromorphic applications

Benedetta Gaggio[1], Atif Jan[1], Moritz Muller[1], Barbara Salonikidou[1], Babak Bakhit[1,2,3], Markus Hellenbrand[1], Giuliana Di Martino[1], Bilge Yildiz[4], Judith L. MacManus-Driscoll[1]*

(1) Department of Materials Science and Metallurgy, University of Cambridge, 27 Charles Babbage Road, Cambridge CB3 0FS, United Kingdom. (2) Electrical Engineering Division, Department of Engineering, University of Cambridge, JJ Thomson Avenue, Cambridge CB3 0FA, United Kingdom. (3) Thin Film Physics Division, Department of Physics (IFM), Linköping University, Linköping SE-58183, Sweden. (4) Laboratory for Electrochemical Interfaces, Massachusetts Institute of Technology, Cambridge, MA, USA.

This supporting information file includes:

1. XRR and AFM investigation of the surface of T-Nb2O5
2. Composition analysis of sub-stoichiometric thin films
3. Electrical performance characterisation
4. Retention comparison between bilayer individual layer devices
5. Spike-Amplitude Dependent Plasticity (SADP) cyclability example and multilevel % change
6. Spike-Time Dependent Plasticity (STDP) voltage profiles
7. In-operando Raman
8. Thermionic emission derivation of Schottky barrier
9. Electrochemical impedance analysis (EIS)
10. EIS derivation of temperature-dependent activation energy

Supplementary 1: XRR and AFM investigation of the surface of T-Nb2O5

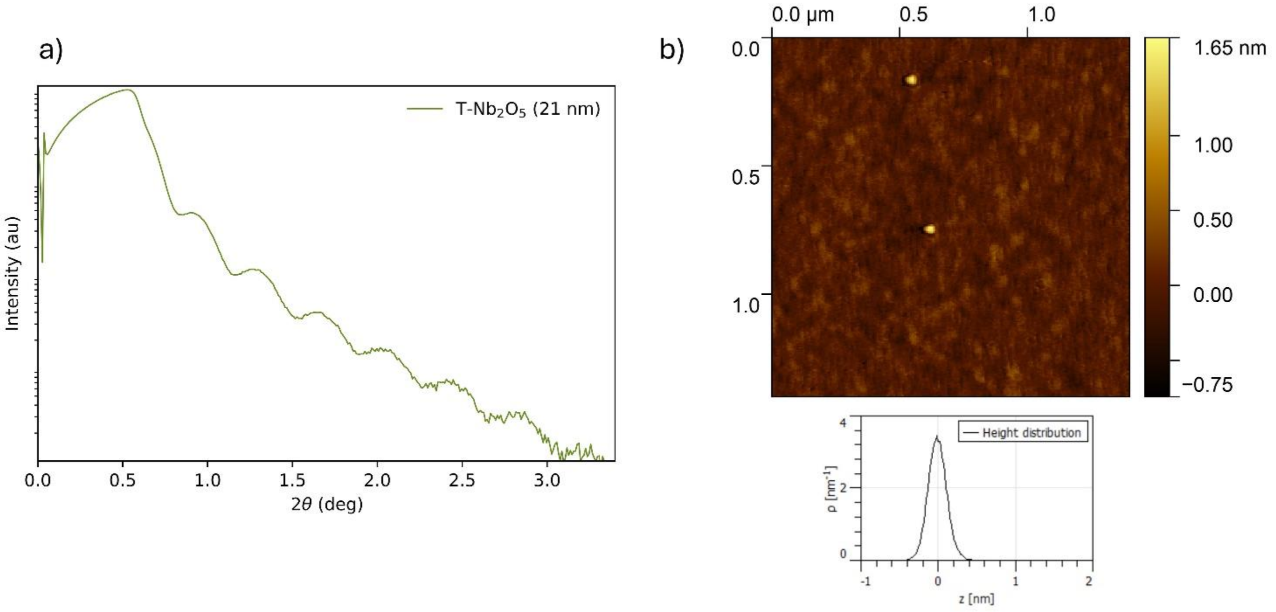

Figure S1: a) X-ray reflectivity spectra of $T\text{-}Nb_2O_5$ (21 nm) single layer on NbSTO, showing excellent low surface roughness character. b) AFM surface morphology study of $T\text{-}Nb_2O_5$ single layer on NbSTO, R_{rms} = 0.126 nm, R_a = 0.096 nm.

Supplementary 2: Composition analysis of substoichiometric thin films

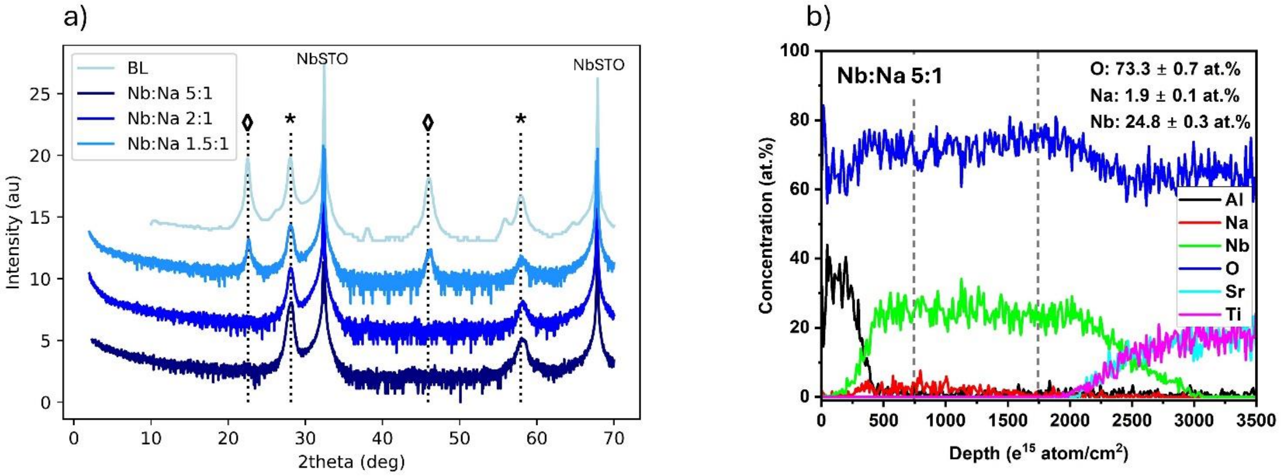

Figure S2: Composition analysis of sub stoichiometric thin films.

a) XRD spectra for Nb_2O_5/$NaNbO_3$ bilayer films deposited with $NaNbO_3$ increasing Nb:Na ratios (from top to bottom); $NaNbO_3$ related-peaks (*denoted by diamond shape*) are only observed in the spectra of samples deposited from targets that are close in stoichiometry (stoichiometry being Nb:Na = 1:1); below Nb:Na 1.5:1, only the Nb_2O_5 peaks are observed (*denoted by asterisk*).

b) ToF-ERDA analysis of bilayer sample deposited employing the Nb:Na 5:1 sample, showing extremely small sodium content, related to contamination rather than $NaNbO_3$, further confirming the requirement of stoichiometric target ratios to deposit $NaNbO_3$ and dope Nb_2O_5.

Supplementary 3: Supplementary electrical performance characterisation

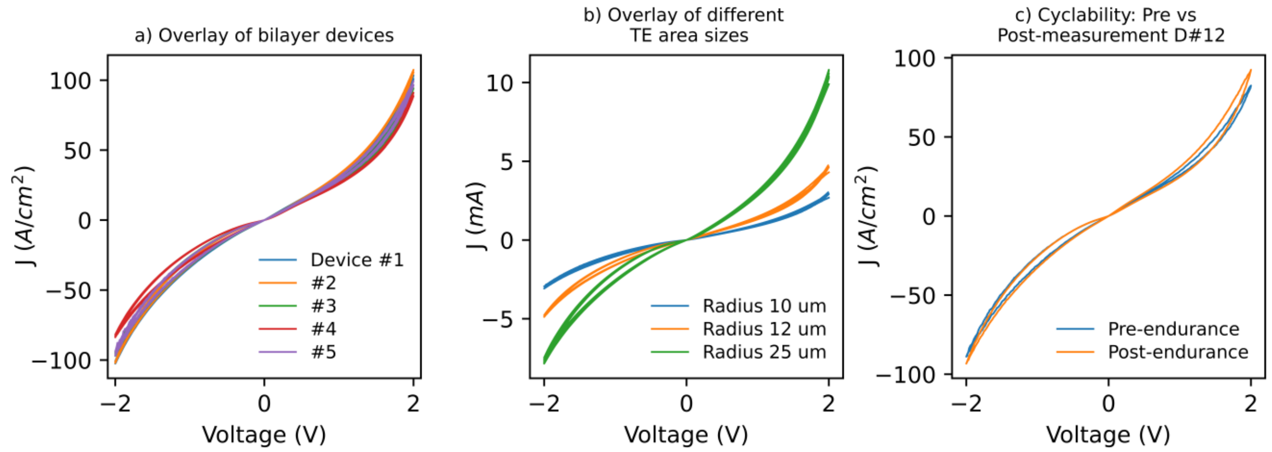

a) IV sweep for different devices showing uniformity across different devices of sample NbSTO/Nb_2O_5/$NaNbO_3$/Cr/Au with electrode radius 25 μm.
b) Current vs voltage plot for different devices of sample NbSTO/Nb_2O_5/$NaNbO_3$/Cr/Au highlighting the current dependence on electrode size.
c) IV sweep of NbSTO/Nb_2O_5/$NaNbO_3$/Cr/Au device before and after 8000 cycles.

Supplementary 4: Retention comparison between bilayer individual layer device

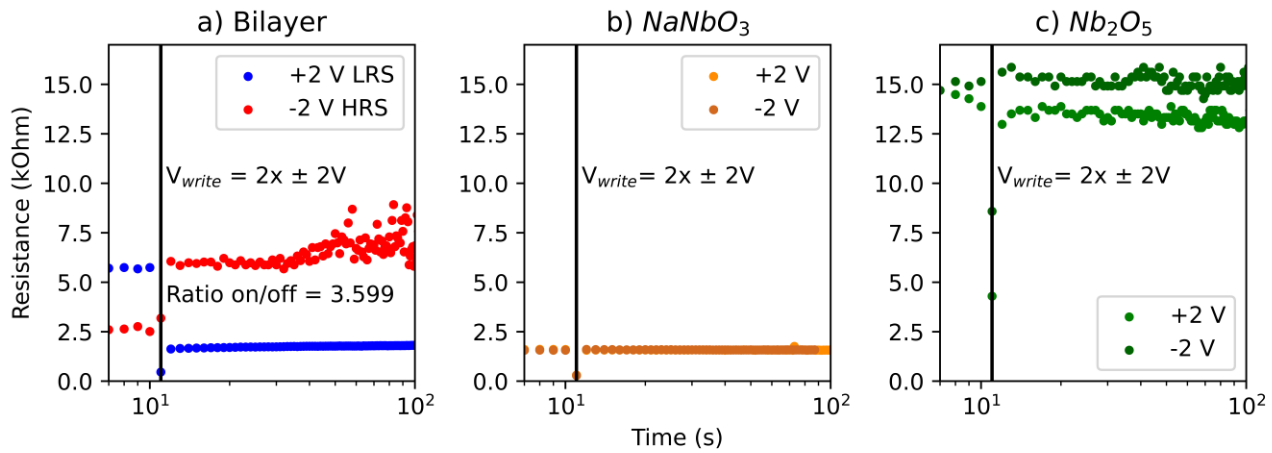

Figure S4: Current density versus time plot to show retention comparison of:

a) NbSTO/Nb_2O_5/$NaNbO_3$ bilayer device (blue: LRS, red: HRS);

b) NbSTO/Nb_2O_5 (orange);

c) NbSTO/$NaNbO_3$ (green). TE = Cr/Au in all cases.

The retention is measured at a V_{read} = 0.1 V, and the devices are set by applying two consecutive V_{write} = ±2 V (line indicating voltage application time). The plots clearly show that only in the bilayer device there is a clear non-volatile resistive switching event taking place after the pulse application, where the device is set into its LRS (blue) and then reset o its HRS (red). Moreover, the application of closely timed double pulse shows an increase in the on/off ratio to a maximum value recorded of 3.599.

Supplementary 5: Spike-Amplitude Dependent Plasticity (SADP) cyclability and multilevel % change

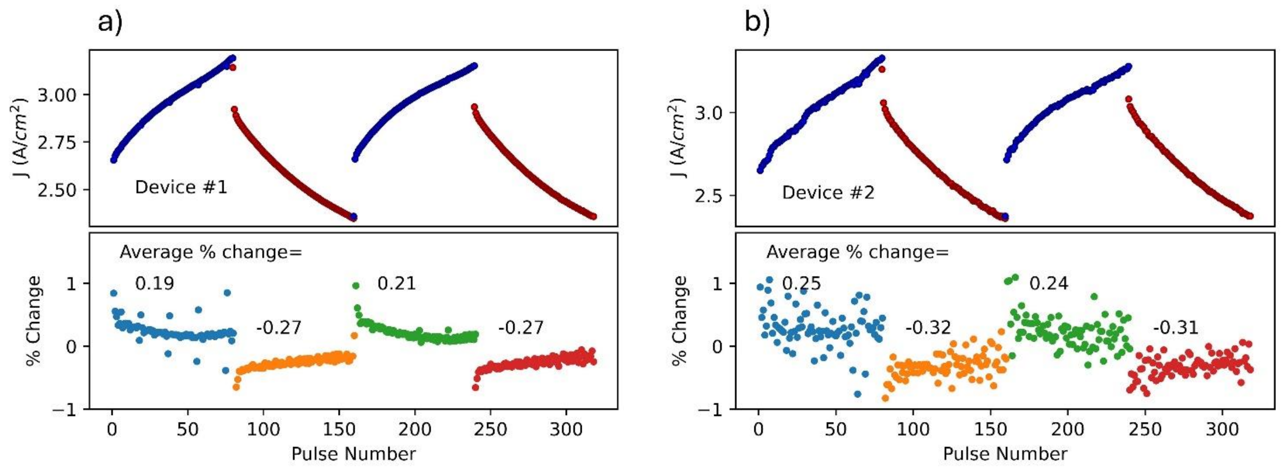

Figure S5: a-b) The SADP experiments are repeated on 2 devices (top panels); the percentage change is calculated by $(J_{i+1} - J_i)/J_i$, where J_i is the current read at 0.1 V after the application of the set/reset pulse, averaged over two read pulses. Device #1 and #2 show good uniformit y in absolute change, though we note that device #2 shows noise characteristics.

Supplementary 6: Plot of voltage profiles for asymmetric pulses in spike-time-dependent plasticity characterisation

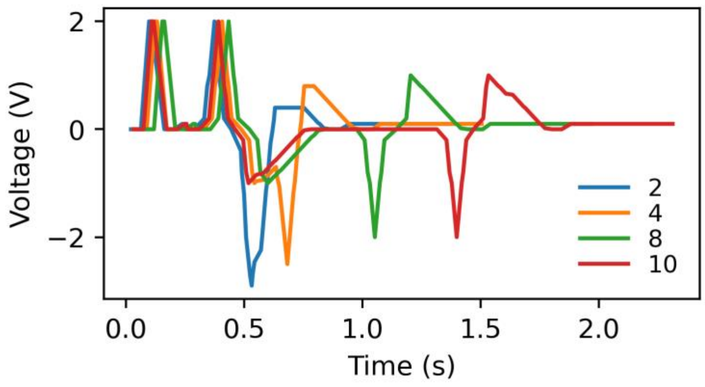

Figure S6: Voltage profiles showing the sum of the pre- and post-synaptic pulse, applied at the TE of the device. Lines 2 to 10 show consecutive measurements of increasing time shift. The initial pulse serves to set the device to its LRS prior to testing the depression of the synaptic weight.

Supplementary 7: In operando Raman spectra

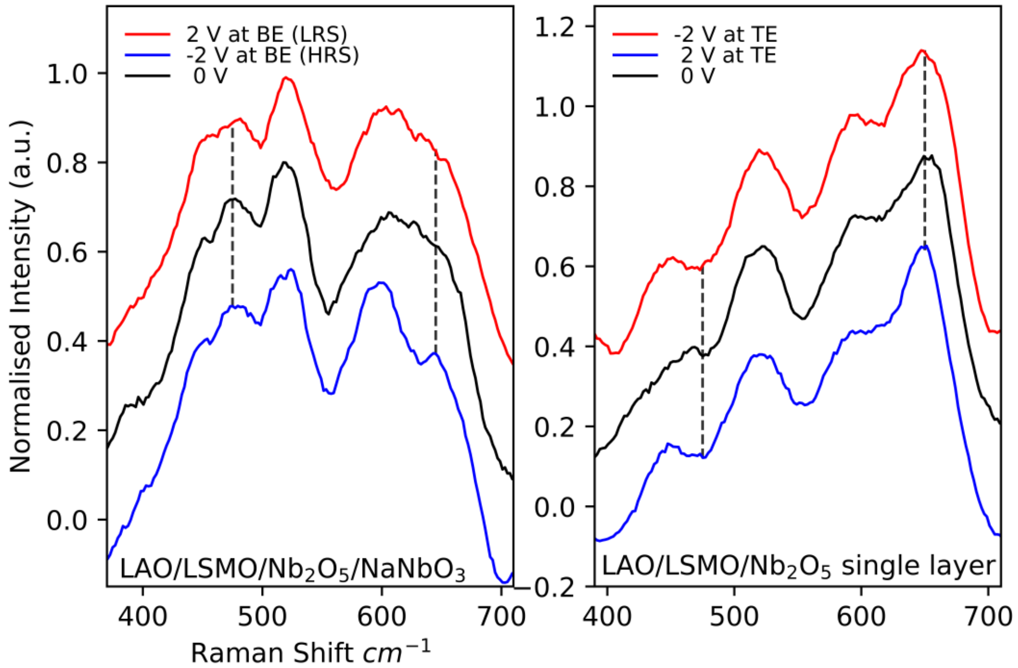

Figure S7: In-operando Raman of:

a) LAO/LSMO/Nb_2O_5/$NaNbO_3$/TE device (same sample reported in Fig. 3(a-b), different device area studied), where the TE is grounded, and the pulse is applied via the BE. I.e., a -ve pulse from the BE sets the device into LRS as Na^+ ions move towards the BE/Nb_2O_5 interface as the dominating barrier, whilst a +ve pulse from the BE resets the HRS by driving Na^+ ions away from the BE/Nb_2O_5 interface. The same relationship between high and low Raman shift region changes in intensity with intercalation is observed with reverse polarity, which confirms the reliability of the measurement.

b) LAO/LSMO/Nb_2O_5/TE single layer device is tested, with pulse applied at the TE. The changes observed in the in-operando Raman spectra of the bilayer device are not seen for the undoped. This test is carried out to further confirm that Raman changes are probing voltage-dependent changes in the Na^+ ionic distribution.
We note that in both S6 (a) and (b), the LAO/LSMO contribution was not subtracted from the overall device signal as it is expected to be also voltage-dependent, and therefore subtraction of the post-mortem signal is not a valid data treatment in the in-operando measurements.

Supplementary 8: Thermionic emission derivation of Schottky barrier

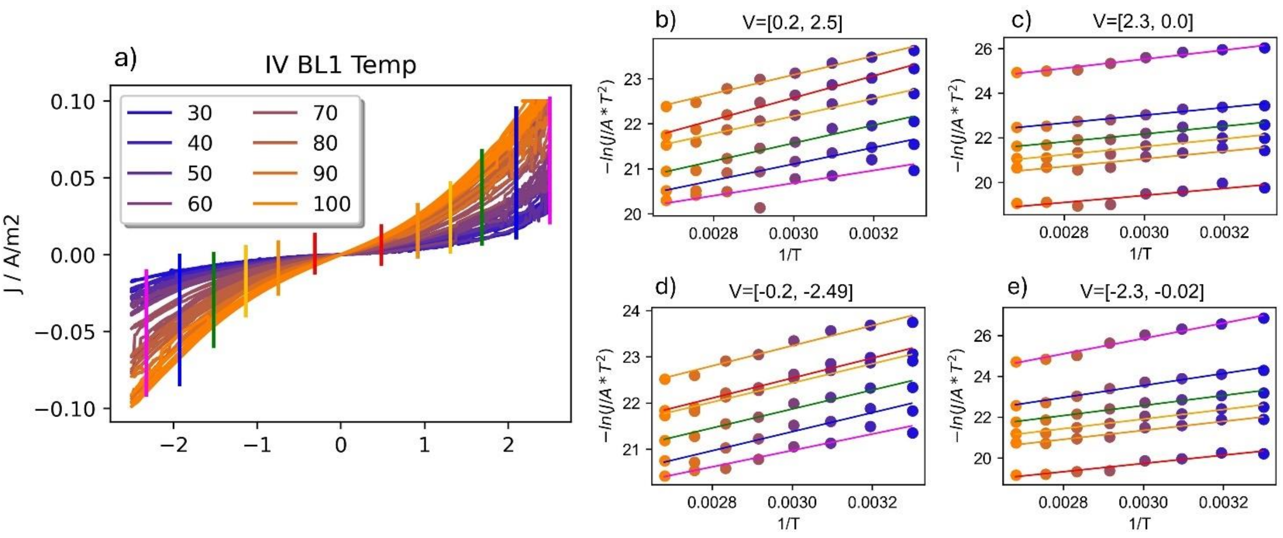

Figure S8:

a) IV scans are collected at increasing temperatures points in the range 30 to 110 ° C (shown by color gradient), followed by fitting with a Richardson plot at voltage ranges: 0.25, 0.50, 1.00, 1.50, 2.00, 2.49 V (represented by vertical lines on IV plot (a)) to extract Schottky barrier values according to Equation S1:

$$J = A^{*}T^{2}\exp\left[\frac{-q}{k_{B}T}\left(\Phi_{B} - \sqrt{\frac{qE}{4\pi\varepsilon_{0}\varepsilon_{op}}}\right)\right]$$

Where J is current density (A/cm^2), $A^{*} = \lambda A_0$is the effective Richardson constant, taken to be $A_0 = 1.2 \times 10^6$ (A/m^2 T^2) and $\lambda = 0.5$, T is temperature (Kelvin), ε_0 is the permittivity of vacuum, Φ_B is Schottky barrier (eV), ε_{op} is the optical dielectric constant, both to be calculated. E is approximated to V/t where $t =$ thickness.

The linear fittings of $\ln\left(\frac{J}{A^{*}T^{2}}\right)$ vs 1/T are shown in the ranges of: b) 0.2 to 2.5 V; c) 2.3 to 0 V; c) -0.2 to -2.5 V; d) -2.3 to -0 V. The SB trends extracted from temperature dependent fits are, respectively: 0.1597, 0.1360, 0.1532, 0.1899 eV, with an overall $\Delta SB_{HRS-LRS}$ = 0.025 ± 0.006 eV.

The findings agree with voltage-dependent fittings. The discrepancy in the absolute values and error values (calculated from standard deviation) is around 50% and may be attributed to the device having more than one mechanism at play, which can't be pictured wholly in this individual mechanism analysis. For example, trap-assisted tunneling (TAT) often plays a role at higher applied voltage, and here the SB is measured up to 2.5 V. Moreover, NbSTO has a temperature-dependent resistance, which may contribute to this percentage change from voltage-dependent derivation.

Supplementary 9: Electrochemical Impedance spectroscopy comparison of bilayer and single layer devices

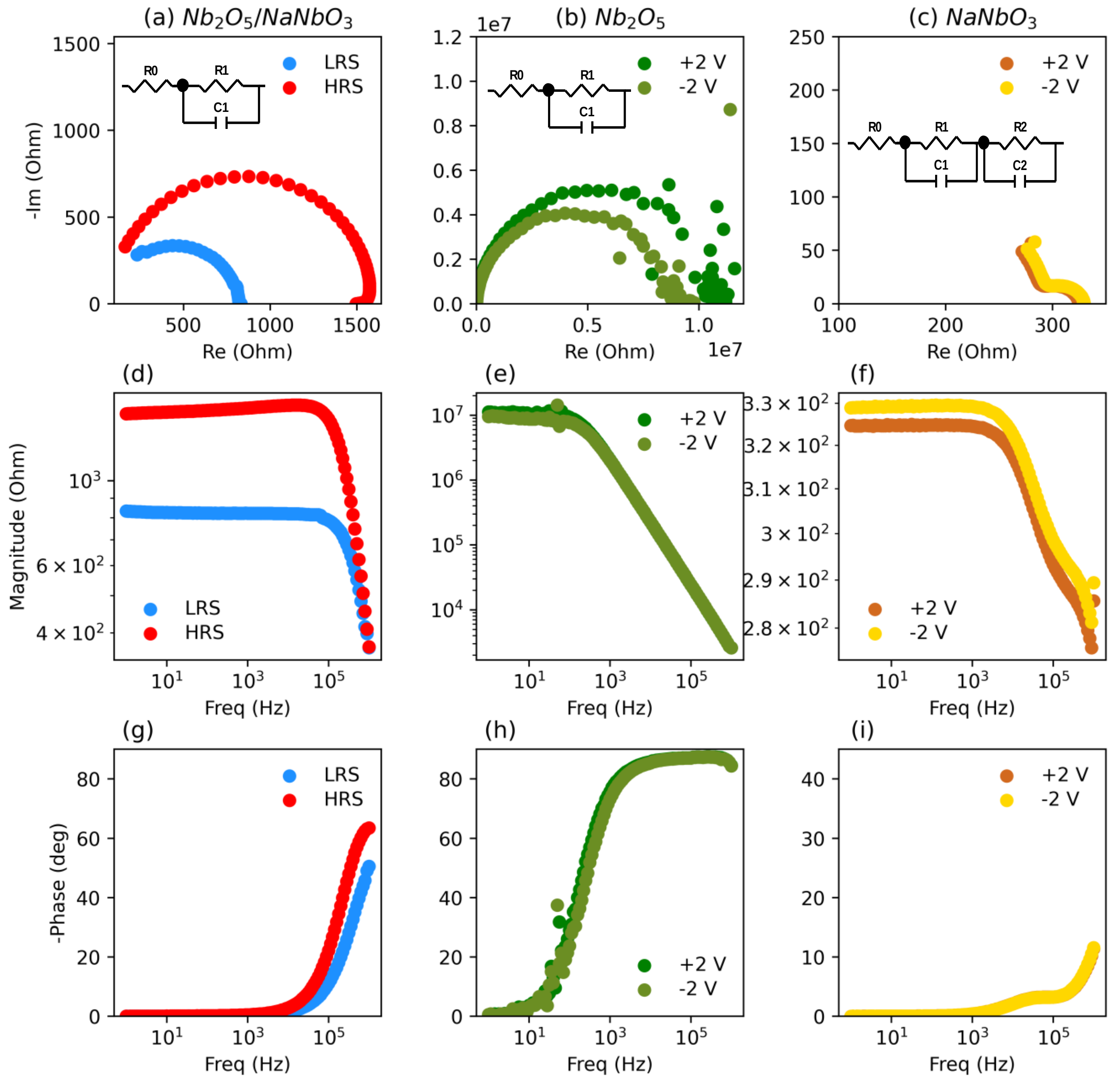

Fig S9: Impedance spectroscopy spectra for NbSTO/Nb_2O_5/$NaNbO_3$/TE device (col 1), NbSTO/Nb_2O_5/TE device (col 2, green), NbSTO/$NaNbO_3$/TE device (col 3, orange): a-c) Nyquist plots with equivalent circuit model employed; d-f) Impedance magnitude (Ohm) vs Frequency (Hz); g-i) Impedance Phase offset (degrees) vs Frequency (Hz).

The EIS spectrum of the bilayer device is compared to the individual layers in Fig. S9. To each device, a ±2 V pulse is applied at the TE before measuring the EIS spectra (1 Hz-1 MHz frequency range, 100 mV amplitude of sinusoidal signal, which matches the V_{read} used thus far).

Firstly, the comparison of the device impedance spectra (of equal overall thickness) clearly indicates that the bilayer device is the only device to show non-volatile changes in its spectra after application of set/reset pulses. The individual layers show negligible change. This was already observed in the dc-voltage retention data in Supplementary note 3, and we relate it to the volatile nature of the single layer devices, versus the non-volatile effects achieved in the bilayer. Secondly, the highly ionic $NaNbO_3$ device (orange) shows the smallest impedance magnitude (around 200 Ohm), with potential high frequency (>1 MHz) contributions that are not visible in the limits of our set up. The leaky nature of this oxygen-deficient layer is therefore further shown by ac-voltage measurements. On the other hand, the T-Nb_2O_5 shows a much larger impedance magnitude (10^7 Ohm). This device has a large capacitive contribution, due to its insulating nature and large dielectric constant. Finally, the bilayer has an impedance magnitude intermediate of its two constituent layers (HRS: $1.5x10^3$ Ohm; LRS: $6x10^2$ Ohm), due to Na-species infiltration. The spectra are fitted with equivalent circuits shown in Fig. S9(a-c), reported in Table S2 below:

Device	R_0 (Ohm)	R_1 (Ohm)	C_1 (F)	R_2 (Ohm)	C_2 (uF)
Bilayer HRS	82	1.5e3	4.3e-10	/	/
Bilayer LRS	165	6.5e2	5.9e-10	/	/
Nb_2O_5	201	8.2e6	7.2e-11	/	/
$NaNbO_3$	142	146	4.9e-10	31	1.5e-7

The C_1 value for the bilayer device are further employed to derive the dielectric constant value. The relevant peak features in the EIS spectra for the HRS and LRS are found in the high frequency region of the spectra, so that comparison to the optical dielectric constant derived from the Schottky fit is appropriate. The geometric capacitance relation is employed to derive dielectric constant according to Equation S2:

$$\kappa = \frac{d}{A\varepsilon_0}C$$

Where C is capacitance (F), A is area (m^2), d is thickness (m), $\varepsilon_o$is vacuum permittivity (F/m) and κ is dielectric constant.

Supplementary 10: Arrhenius temperature dependence

The device conductivity is governed by the Schottky conduction barriers in the bilayer band alignment, which in turn relate to the complex interfacial effects taking place at each barrier due to voltage-controlled motion of charge carrier Na^+. The ionic species diffusion can be described by the Arrhenius equation, which considers the temperature-dependence of the ionic species motion. The temperature-dependent activation energy of diffusion can therefore be extrapolated from Equation S3:

$$\ln(\sigma) = A + \frac{-E_A}{k_B} \times \frac{1}{T}$$

Where σ is the conductivity, A is a pre-exponential factor constant, E_A is the activation energy for ionic charge carrier diffusion (meV), k_B is the Boltzmann constant, and T is

temperature (Kelvin). The impedance spectra of each device investigated is measured at various temperatures (in the range 20 to 70 °C, at 5 °C intervals). The spectra are fitted employing an equivalent circuit (shown in Fig. S9) to measure the fitted conductivity. The activation energy is then extrapolated from the slope of the linear fits of conductivity versus inverse temperature (Equation S3) plots.
